# The Severity of Diabetic Retinopathy Corresponds with Corneal Nerve Alterations and Ocular Discomfort of the Patient

**DOI:** 10.3390/ijms25116072

**Published:** 2024-05-31

**Authors:** Anna Machalińska, Agnieszka Kuligowska, Alicja Ziontkowska-Wrzałek, Beata Stroynowska, Ewa Pius-Sadowska, Krzysztof Safranow, Jan Machaliński, Katarzyna Mozolewska-Piotrowska, Bogusław Machaliński

**Affiliations:** 1 First Department of Ophthalmology, Pomeranian Medical University, Al. Powstańców Wielkopolskich 72, 70-111 Szczecin, Poland; agnieszka.kaleta91@gmail.com (A.K.); aziontkowskawrzalek@gmail.com (A.Z.-W.); b.stroynowska@spsk2-szczecin.pl (B.S.); janekmach89@gmail.com (J.M.); katarzyna.mozolewska.piotrowska@pum.edu.pl (K.M.-P.); 2 Department of General Pathology, Pomeranian Medical University, Al. Powstańców Wielkopolskich 72, 70-111 Szczecin, Poland; ewapius@wp.pl (E.P.-S.); boguslaw.machalinski@pum.edu.pl (B.M.); 3 Department of Biochemistry and Medical Chemistry, Pomeranian Medical University, Al. Powstańców Wielkopolskich 72, 70-111 Szczecin, Poland; chrissaf@mp.pl

**Keywords:** BDNF, confocal microscopy, corneal innervation, corneal sensitivity, cytokines, diabetic retinopathy, neurotrophins, OSDI, tear film, type 2 diabetes mellitus

## Abstract

Diabetic retinopathy (DR) remains the leading cause of blindness in the working-age population. Its progression causes gradual damage to corneal nerves, resulting in decreased corneal sensitivity (CS) and disruption of anterior-eye-surface homeostasis, which is clinically manifested by increased ocular discomfort and dry eye disease (DED). This study included 52 DR patients and 52 sex- and age-matched controls. Ocular Surface Disease Index (OSDI) survey, tear film-related parameters, CS, and in vivo corneal confocal microscopy (IVCM) of the subbasal plexus were performed. Furthermore, all patients underwent tear sampling for neurotrophin and cytokine analysis. OSDI scores were greater in DR patients than in controls (*p* = 0.00020). No differences in the Schirmer test score, noninvasive tear film-break-up time (NIBUT), tear meniscus or interferometry values, bulbar redness, severity of blepharitis or meibomian gland loss were found. In the DR group, both the CS (*p* < 0.001), and the scotopic pupil diameter (*p* = 0.00008) decreased. IVCM revealed reduced corneal nerve parameters in DR patients. The stage of DR was positively correlated with the OSDI (Rs = +0.51, 95% CI: + 0.35–+0.64, *p* < 0.001) and negatively correlated with IVCM corneal nerve parameters and scotopic pupillometry (Rs = −0.26, 95% CI: −0.44–−0.06, *p* = 0.0097). We found negative correlations between the OSDI and IVCM corneal innervation parameters. The DR group showed lower tear film-brain-derived neurotrophic factor (BDNF) levels (*p* = 0.0001) and no differences in nerve growth factor (NGF)-β, neurotrophin (NT)-4, vascular endothelial growth factor (VEGF), interleukin (IL)-1β, IL-4, IL-5, IL-6, or IL-12 concentrations. Tumor necrosis factor (TNF)-α, IL-2, IL-8, IL-10, granulocyte macrophage colony-stimulating factor (GM-CSF), and interferon (IFN)-γ levels were decreased among patients with DR. Corneal innervation defects have a direct impact on patients’ subjective feelings. The evolution of DR appears to be associated with corneal nerve alterations, emphasizing the importance of IVCM.

## 1. Introduction

The cornea represents the major element of the optical system. Due to its dense innervation, it plays a crucial role in tear secretion and maintaining the proper composition and function of the tear film. Thus, corneal sensory nerves are the major determinants of sustained ocular surface homeostasis [1].

Anterior-eye-surface homeostasis is retained when the cornea, its surface, tear film, and other elements of the anterior eye remain in volatile balance, enabling it to respond to external stressors in an autoregulating manner without inducing excessive inflammation, pain, or other pathological events. Dry eye disease (DED) can lead to tear film-homeostasis loss and ocular symptoms, with tear film instability and hyperosmolarity, ocular surface inflammation and injury, and neurosensory abnormalities all playing etiological roles [2,3].

Although diabetic retinopathy (DR) is the most prevalent and well-known ophthalmic consequence, diabetes mellitus (DM) can also have substantial effects on the ocular surface [4]. Recently, there has been much interest in examining DM-induced changes in the human cornea [5]. Chronic hyperglycemia in diabetic patients can cause damage to nerve fibers, leading to reduced corneal nerve fiber density, attenuated corneal sensitivity (CS), and diabetic corneal neuropathy (DCN) [6,7].

Sustained hyperglycemia and dyslipidemia in type 2 diabetes mellitus (T2DM) promote neuronal degeneration through accumulating advanced-glycation end products, increasing polyol pathway activity, activating the proteinase K pathway, and causing oxidative stress [8].

Interestingly, several investigations have shown that alterations in corneal nerve fibers are linked to the development of DR [1].

Indeed, DR is a neurovascular disease. Numerous studies indicate that neurons are vulnerable to injury shortly following the onset of DM, before any vascular damage occurs. In the diabetic retina, apoptosis of neurons and activation of glial cells can generate metabolic dysregulation, resulting in oxidative stress and damage to the retina cells [9].

The clinical symptoms in diabetic patients with DCN are photophobia, eye irritation, or pain [10]. Diabetic corneal neuropathy affects 47–64% of DM patients and is largely underdiagnosed [11].

Our objective was to investigate the potential correlation between DCN and DR in individuals with T2DM.

Currently, there is a gap in the literature regarding the impact of DCN on DED symptoms. Moreover, there still is a lack of data evaluating corneal nerve alterations in DR patients in both subjective and objective ways, employing both corneal sensory testing and in vivo corneal confocal microscopy (IVCM), as well as data on their relationships with the levels of neurotrophic factors in tears.

To objectively assess the structural condition of the subbasal nerve plexus (SNP), we applied in vivo confocal imaging analysis. In addition, we characterized the relationship between ocular diabetic retinopathy and neuropathy and both objective and subjective dry eye symptoms. To determine the local inflammation status, we analyzed a panel of selected inflammatory cytokines in tears. We also evaluated the concentrations of selected neurotropic factors and their relationships with ocular parameters. To our knowledge, such comprehensive data on corneal innervation status and eye surface homeostasis in DR patients and their correspondence with the inflammatory state and neurotropic factor levels in tears have not been previously investigated.

## 2. Results

### 2.1. Study Groups do Not Differ in Their Clinical Characteristics

A total of 98 eyes of 52 patients with DR were included. As a control group, we enrolled 52 (103 eyes) healthy subjects with no history of DM. The study groups were matched for age (*p* = 0.17) and sex (*p* = 0.07). The groups did not differ in body mass index (BMI) measurements, occurrence of hypertension, or smoking status. Accordingly, there were no significant differences between the groups in intraocular pressure (IOP) values, the incidence of cataract surgery during anamnesis, the prevalence of glaucoma, or anti-glaucomatous eyedrop use. Table 1 summarizes the clinical characteristics of the DR patients and the controls (Table 1).

The mean duration of diabetes in DR patients was 2031 months (ranging from 1 to 480 months), and 69% of patients required insulin medication. A total of 7 patients (13.46%) were diagnosed with diabetic foot disease, 3 patients (5.76%) were diagnosed with diabetic kidney disease, and 12 patients (23.08%) were diagnosed with diabetic neuropathy. Of the 98 eyes examined, 14 were assigned to subgroup 1 based on disease severity as determined by the International Clinical Diabetic Retinopathy Disease Severity Scale, 17 were assigned to subgroup 2, 25 were assigned to subgroup 3, and 42 were assigned to subgroup 4. Furthermore, of the 98 eyes examined, 85 were diagnosed with diabetic macular edema (DME) (12 were Grade 1, 19 were Grade 2, and 52 were Grade 3), and 25 underwent panretinal photocoagulation (PRP). Analysis of the patients’ medical histories revealed that among the 98 eyes analyzed, 30 were treated with anti–vascular endothelial growth factor (anti-VEGF) intravitreal injections. The mean number of injections received was 5.5, with a maximum of 16 and a minimum of 1 injection per eye.

### 2.2. Tear Film-Related Parameters Are Similar in DR Patients and Healthy Individuals

Next, we focused on evaluating self-reported dry eye symptoms and tear film characteristics in the study subjects. We found that Ocular Surface Disease Index (OSDI) scores were significantly greater in diabetic patients than in controls (mean: 29.46 vs. 11.67 points; *p* < 0.001). The difference remained significant in the multivariate analysis performed in the general linear model after adjustment for age and sex (*p* = 0.00020). Most importantly, ocular symptom scores were strongly positively correlated with the severity of DR (Rs = +0.51, 95% CI: +0.35–+0.64, *p* < 0.000001), and patients with proliferative diabetic retinopathy (PDR) had higher OSDI values than did those with nonproliferative diabetic retinopathy (NPDR) (median: 40 vs. 11.5, respectively; *p* < 0.001). Accordingly, the OSDI was significantly greater in patients who underwent PRP than in controls (median: 43 vs. 23, respectively; *p* < 0.001). We did not observe any significant differences in the Schirmer test values, noninvasive tear film break-up time (NIBUT), bulbar redness, severity of blepharitis, tear meniscus, or interferometry results between the DR and control groups. Similarly, no differences were found in meibomian gland loss between the groups (*p* = 0.92) (Table 2).

### 2.3. Subjective and Objective Corneal Innervation Parameters Are Lower in DR Patients Than in Controls

Because available data suggest that DR may be associated with corneal nerve loss and compromised corneal nerve function, we performed a comprehensive quantitative analysis of corneal nerve morphology and evaluated corneal sensation alterations. We found that the mean CS was significantly lower in the DR group than in the control group (median: 5.5 vs. 6, respectively; *p* < 0.001). Accordingly, the CSs detected in five different locations of the cornea—the superior-nasal (SN), superior-temporal (ST), inferior-nasal (IN) and inferior-temporal (IT) quadrants—and the center of the cornea (CC) appeared to be significantly lower in the DR group than in the control group. Interestingly, central CS negatively correlated with OSDI, indicating that low CS is related to high symptom scores (Rs = −0.26, 95% CI: −0.44–−0.06, *p* = 0.011).

**Figure 1:**
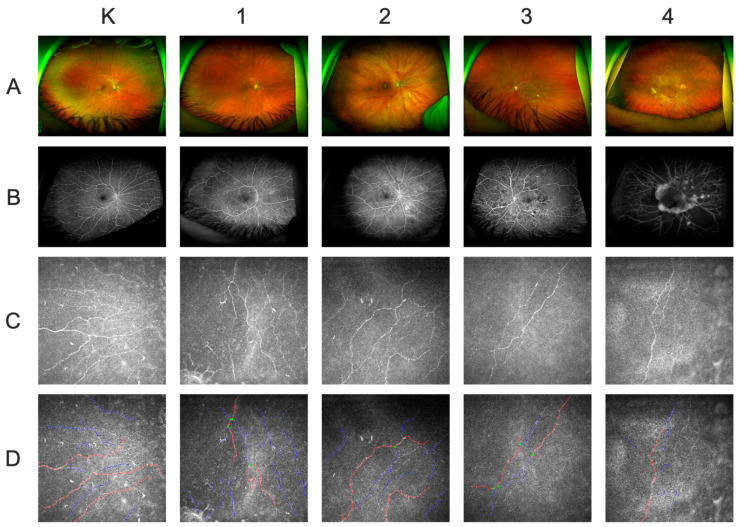
(**A**): Ultra-wide-field fundus photography images (angular range: 200 degrees; resolution: 14 μm); (**B**): corresponding fluorescein angiography images (angular range: 200 degrees; resolution: 14 μm); (**C**): corresponding in vivo corneal confocal microscopy images of the subbasal nerve plexus (800× magnification; field of view: 400 × 400 μm); (**D**): corresponding in vivo corneal confocal microscopy images of the subbasal nerve plexus using ACCMetrics software (https://sites.manchester.ac.uk/ccm-image-analysis/; marked red—main fibers, marked blue—branches, marked green—branching points) (800× magnification; field of view: 400 × 400 μm); **K**—a healthy control; **1**—Stage 1: mild nonproliferative diabetic retinopathy (NPDR); **2**—Stage 2: moderate NPDR; **3**—Stage 3: severe NPDR; **4**—Stage 4: proliferative retinopathy (PDR).

To objectively quantify the structural status of the SNP, we utilized in vivo confocal microscopy analysis. The IVCM analysis of subbasal nerves is presented in Table 3. The corneal nerve fiber density (CNFD), corneal nerve fiber length (CNFL), corneal nerve branch density (CNBD), corneal nerve fiber area (CNFA), corneal nerve fiber width (CNFW), corneal nerve fractal dimension (CNFrDim), and corneal nerve fiber reflectivity (CNFRe) values were significantly lower in DR patients than in controls. All these associations except for the CNFW (*p* = 0.075) remained statistically significant after adjusting for age and sex in multiple linear regression modelling (*p* < 0.05). Most importantly, the stage of DR was negatively correlated with all analyzed corneal nerve parameters (Rs = −0.30, 95% CI: −0.47–−0.11, *p* = 0.0029 for CNFD; Rs = −0.26, 95% CI: −0.44–−0.06, *p* = 0.0090 for CNFL; Rs = −0.28, 95% CI: −0.45–−0.09, *p* = 0.0053 for CNBD; Rs = −0.22, 95% CI: −0.40–−0.02, *p* = 0.032 for CNFA; Rs = −0.20, 95% CI: −0.38–−0.002, *p* = 0.046 for CNFW; Rs = −0.23, 95% CI: −0.41–−0.03, *p* = 0.023 for CNFrDim; Rs = −0.25, 95% CI: −0.43–−0.05, *p* = 0.014 for CNFTo; Rs = −0.27, 95% CI: −0.44–−0.08, *p*= 0.0083 for CNFRe). Accordingly, patients with proliferative retinopathy had lower CNFD, CNFL, CNBD, CNFW, CNFrDim, corneal nerve fiber tortuosity (CNFTo), and CNFRe values than did those with nonproliferative retinopathy (median: 6.2 vs. 10.4 no./mm^2^, respectively, *p* = 0.003 for CNFD; 5.4 vs. 7.4 mm/mm^2^, *p* = 0.02 for CNFL; 4.2 vs. 12.5 no./mm^2^, *p* = 0.004 for CNBD; 0.0223 vs. 0.0216 mm/mm^2^, *p* = 0.05 for CNFW; 1.3 vs. 1.4, *p* = 0.02 for CNFrDim; 2 vs. 2, *p* = 0.02 for CNFTo; median 2 vs. 3, *p* = 0.001 for CNFRe). This finding implies that patients with more advanced retinopathy on the fundus exhibit more severe corneal innervation abnormalities (Figure 1). Importantly, we found negative correlations between the OSDI and the CNFD (Rs = −0.29, 95% CI: −0.46–−0.10, *p* = 0.0039), CNFL (Rs = −0.23, 95% CI: −0.41–−0.03, *p* = 0.023), CNBD (Rs = −0.28, 95% CI: −0.45–−0.09, *p* = 0.0057), and CNFRe (Rs = −0.43, 95% CI: −0.58–−0.25, *p* = 0.000011), indicating a direct relationship between altered corneal innervation and high symptom scores (Table 3).

### 2.4. Scotopic Pupil Diameter Is Smaller in the DR Group Than in Healthy Individuals

Pupil diameter (PD) has been used as a diagnostic indicator for a wide range of autonomic nervous system dysfunctions. Thus, we analyzed mesopic, scotopic, and photopic pupil diameters. We found that the scotopic pupil diameter was significantly smaller in the diabetic group than in the control group (median: 3.3 mm vs. 4.15 mm, respectively; *p* < 0.001). The difference remained significant in the multivariate analysis performed in the general linear model after adjustment for age and sex (*p* = 0.00008). More importantly, a significant negative correlation between the stage of DR and scotopic pupillometry was found (Rs = −0.26, 95% CI: −0.44–−0.06, *p* = 0.0097). Accordingly, patients with PDR had smaller scotopic pupil sizes than did those with NPDR (median: 3.1 vs. 3.55 mm, respectively; *p* = 0.005), and patients who underwent PRP had significantly lower scotopic pupil sizes than controls (median: 2.9 vs. 3.5 mm, respectively; *p* = 0.003). No significant differences in the fotopic pupil diameters were found between the groups (median: 2.4 mm in the DR group vs. 2.4 mm in the control group, *p* = 0.77). Accordingly, no differences in mesopic pupil diameter were observed (median: 1.6 mm in the DR group vs. 2.6 mm in the control group, *p* = 0.67).

As altered blinking behavior might reflect reduced CS, we analyzed the blink response in DR patients and controls. We found that the interblink interval (IBI) was greater in the diabetic group than in the control group (median: 3.8 s vs. 2.9 s, respectively; *p* = 0.04). The difference lost significance in the multivariate analysis performed in the general linear model after adjustment for age and sex (*p* = 0.17).

### 2.5. Levels of Selected Pro- and Anti-Inflammatory Cytokines in the Tears of DR Patients Are Either Lower or the Same as Those in the Control Group

Then, we aimed to investigate the concentrations of selected cytokines in tear film samples collected from patients and controls. The tear film concentrations of tumor necrosis factor (TNF)-α, interleukin (IL)-2, IL-8, IL-10, granulocyte macrophage colony-stimulating factor (GM-CSF), and interferon (IFN)-γ were significantly lower in patients with DR than in control participants (Table 4). Accordingly, no differences in the tear film concentrations of vascular endothelial growth factor (VEGF), IL-1β, IL-4, IL-5, IL-6, or IL-12 were found between the study groups.

Interestingly, we found a positive correlation between the mean CS concentration and the TNF-α, VEGF, IL-2, IL-4, GM-CSF, and IFN-γ concentrations in the tear film (Rs = +0.25, 95% CI: +0.05–+0.43, *p* = 0.015; Rs = +0.20, 95% CI: 0.00–+0.38, *p* = 0.050; Rs = +0.26, 95% CI: +0.06–+0.44, *p* = 0.012; Rs = +0.20, 95% CI: 0.00–+0.38, *p* = 0.050; Rs= +0.38, 95% CI: +0.20–+0.54, *p* = 0.00014; Rs = +0.21, 95% CI: +0.01–+0.39, *p* = 0.037, respectively).

### 2.6. Neurotrophic Factor BDNF Levels in Tears of DR Patients Are Lower Than in the Control Group

The tear film levels of brain-derived neurotrophic factor (BDNF) were significantly lower in the DR group than in the control group (median: 1.43 vs. 2.17 pg/mg, respectively; *p* = 0.0001). The difference remained significant in the multivariate analysis performed in the general linear model after adjustment for age and sex (*p* = 0.0028). No differences in the tear film concentrations of nerve growth factor (NGF)-β (median was 0.97 pg/mg in the DR group vs. 0.90 pg/mg in the control group, *p* = 0.10) or neurotrophin (NT)-4 (median was 6.98 pg/mg in the DR group vs. 6.15 pg/mg in the control group, *p* = 0.06) were detected between the study groups (Table 5).

## 3. Discussion

In this study, we assessed the relationship between DR and patients’ self-reported dry eye symptoms. We revealed that the OSDI of DR patients was considerably greater than that of the control group. Furthermore, our research demonstrated that ocular symptom scores strongly positively correlated with the severity of DR. Our results are consistent with previous data [12]. Han JX et al. reported that a greater degree of retinopathy corresponds to greater ocular surface discomfort. Their study consisted of 129 type 2 DM patients divided into four groups. The overall OSDI score of the PDR group was double that of the NPDR group [13]. This finding aligns with our investigations in which patients with PDR had higher OSDI values than those with NPDR.

DED is a chronic inflammatory condition that involves multiple ocular surface structures, such as the cornea, conjunctiva, lacrimal, and meibomian glands. Thus, any dysregulation of ocular surface homeostasis causes an increase in tear break-up time, increased osmolality and interferometry changes, and a reduction in tear film volume, all of which are indicators of DED. Several studies have documented an increased prevalence of dry eye symptoms in DM patients compared to healthy controls [14].

Our goal was to determine whether the study participants displayed dry eye-driven ocular surface abnormalities. Interestingly, our study revealed no significant differences in Schirmer’s test, NIBUT, interferometry, or tear meniscus height between the DR and control groups. Similarly, we found no differences in blepharitis exacerbation or meibomian gland loss. These findings are remarkable, primarily because one may hypothesize that patients with DR who have a high OSDI score should additionally exhibit objective symptoms of dry eyes.

The findings in the literature are not uniform. Although most authors have reported a connection between DR and dry eye syndrome, identified by its objective symptoms [15,16], certain reports have shown opposing findings. Goebbels M et al. reported that the tear film break-up time did not differ between the DR group and the nondiabetic control group [17]. Li HY et al. reported comparable Schirmer values between DM patients and controls. Moreover, there were no significant differences in the Schirmer test score between DM patients without retinopathy and DM patients with retinopathy [18]. A few studies have investigated the relationship between DED and the stages of DR. Although some studies have confirmed this association [15], other studies have found no correlation between the DR classification and the incidence of DED [11].

To thoroughly define the underlying inflammatory tear film profile, we molecularly stratified the DR and control groups. There are numerous data indicating that dysregulation of local immune/inflammatory regulatory pathways is a key factor in DED pathogenesis [19]. A meta-analysis performed by Roda M et al. revealed increased levels of the tear-inflammatory mediators IL-1β, IL-6, IL-8, IL-10, IFN-γ, and TNF-α in the tears of DED patients compared to those in the tears of age-matched controls [20]. Consequently, a study performed by Massingale ML et al. revealed statistically significant increases in the concentrations of IL-2, IL-4, IL-6, IL-10, TNF-α, IL-1β, and IL-5 in the tears of DED patients compared to those in the tears of normal controls [21].

According to our findings, patients with DR had cytokine levels that were either much lower than (TNF-α, IL-2, IL-8, IL-10, GM-CSF, and IFN-γ) or the same as (VEGF, IL-1β, IL-4, IL-5, IL-6, and IL-12) those in the control group. This indicates the absence of local immune system activation within the ocular surface in DR patients.

Numerous studies have compared the levels of cytokines in the tears of individuals with DR to those in the tears of healthy controls. The outcomes exhibit inconsistencies. Although the levels of some cytokines were greater in the tears of the DR group, the levels of certain cytokines did not significantly change. Moreover, the amount of cytokines in tears varies depending on the stage of DR and does not necessarily increase as the retinopathy stage increases [22,23].

The available data suggest that DR may be associated with corneal nerve loss and impaired function. Using a Cochet-Bonnet aesthesiometer, Salami MO et al. evaluated CS in 120 diabetic patients and 120 age- and sex-matched nondiabetic patients. According to their findings, diabetic patients with retinopathy had considerably reduced CS in comparison to diabetic patients without retinopathy. Additionally, as DR progresses, so does the extent of corneal sensory loss [24]. According to a meta-analysis conducted by Lv H et al., the CS of patients with PDR was considerably lower than that of patients without DR or with NPDR [25]. Our research revealed that the CS concentration appeared to be significantly lower in the DR group than in the control group. Importantly, CS negatively correlated with OSDI, indicating that low CS is related to high symptom scores. Our findings are in line with those of Rahman EZ et al., who reported that high OSDI is linked to decreased CS [26].

Because of the subjective nature of CS readings, we objectively assessed the structural condition of the SNP via in vivo confocal imaging analysis.

IVCM is a new and simple technology that measures small nerve fibers in a noninvasive and repetitive manner. Compared to conventional techniques, IVCM is fast and precise and provides a more dynamic view of the microscopic layers of the cornea. This high-resolution method of corneal examination provides an objective and highly sensitive way to measure corneal nerve fiber characteristics in diabetic patients [8,27]. Several studies have confirmed the usefulness of IVCM for analyzing the corneal SNP in patients with DM [5,11].

In our study, the CNFD, CNFL, CNBD, CNFA, CNFW, CNFrDim, and CNFRe values were significantly lower in DR patients than in controls. Additionally, patients with PDR had lower CNFD, CNFL, CNBD, CNFW, CNFrDim, CNFTo, and CNFRe values than did those with NPDR. Our results are consistent with other reports [5,11]. Our findings showed a negative association between OSDI and CNFD, CNFL, CNBD, and CNFRe, indicating a link between a high OSDI symptom score and altered corneal innervation. Our results corroborate those of the study by Qin G et al., who demonstrated that corneal nerve loss was associated with ocular pain and decreased CS in diabetic patients [28].

Interestingly, we found that the IBI was greater in the diabetic group than in the control group. Our findings align with a study published by Inoue K et al. on 163 individuals with T2DM and 76 individuals without this condition [29]. Interestingly, the IBI has been reported to be significantly shorter for dry eye patients than for healthy controls [30]. This could imply that the elevated IBI in diabetic individuals in our study was related to impaired innervation and was not related to DED symptoms.

We discovered that the scotopic PD of the diabetic group was considerably lower than that of the control group. Our results are partially consistent with a study conducted by Erdem S et al., who assessed 40 patients with DM with NPDR, 40 patients with DM without DR and 40 healthy controls. In their results, scotopic PD and low photopic PD were significantly lower in the NPDR group than in patients with DM without DR [31]. After analyzing 133 participants, Kızıltoprak H et al. reported that the duration of DM was inversely and moderately correlated with the scotopic PD [32]. This finding is in agreement with our findings, which showed a substantial inverse relationship between scotopic pupillometry and the stage of DR. Interestingly, their study also revealed a potential correlation between diabetic autonomic neuropathy and DR patients, with pupillometry serving as a helpful screening method to identify the condition.

To comprehensively assess neurosensory homeostasis of the eye surface, we analyzed the levels of selected neurotrophic factors in the tears of patients with DR and healthy controls. We found that DR patients had reduced levels of secretory BDNF. Research indicates that BDNF influences cell survival, proliferation, and plasticity; controls neural development; modulates synaptogenesis; and plays a significant role in the synaptic connections of a many neurons. [9,33]. BDNF is present in both the corneal epithelium and stromal keratocytes and is thought to originate from corneal sensory neurons. Research indicates that BDNF can induce the growth and regeneration of both peripheral and central nerves. Additionally, stimulating other neurotrophins and neurotrophic factors can aid in neurite regrowth.

Consistent with our data, the study of Bakhritdinova F et al. revealed a link between the progression of DR and lower BDNF levels in tears. Indeed, BDNF levels in the serum of diabetic patients have been shown to be lower in numerous investigations. After testing 114 people, Kaviarasan K et al. concluded that low serum BDNF is linked to the development of DR and may be a risk factor for DR in individuals with T2DM [34]. Furthermore, an examination of 174 patients with T2DM and 84 healthy people revealed that BDNF was considerably lower in patients with DM than in controls. Additionally, BDNF appears to be a useful diagnostic marker for the detection of DR at early stages [35]. Interestingly, retinal neurodegeneration in DR patients is thought to be linked to a steady decrease in BDNF expression [35,36]. Sun Q et al. investigated 258 subjects and reported that BDNF levels are involved in the progression of diabetic peripheral neuropathy (DPN) [37]. The aforementioned research indicates that a low serum level of BDNF may be a risk factor for diabetic neuropathic complications. To the best of our knowledge, this is the first study examining the relationship between BDNF tear levels and corneal sensation in individuals suffering from diabetic retinopathy.

Interestingly, we found no statistically significant differences in the tear film concentrations of NGF-β and NT-4 between the study groups. Nevertheless, NGF-β levels in tears were marginally higher in the DR group than in the control group. Furthermore, the NT-4 levels were higher, with borderline significance. To the best of our knowledge, no studies have reported decreased NGF and NT-4 levels in patients with diabetic retinopathy. Park KS et al. evaluated NGF levels in the tears of 254 DR patients and 71 nondiabetic controls. PDR patients were shown to have greater levels of tear NGF than did NPDR patients and nondiabetic controls [38]. Similar results were reported by Lee H et al. in a study involving 40 patients with NPDR, 29 patients with PDR, and 23 age- and sex-matched controls. Tears and serum NGF concentrations were significantly greater in PDR patients than in NPDR patients and controls [39].

Research findings indicate that NGF production is upregulated under hyperglycemic conditions [38]. Thus, elevated NGF levels could be the result of increased sugar concentrations in bodily fluids.

Another hypothesis is that a damaged ocular surface and decreased corneal sensitivity may act as drivers of NGF gene expression.

We acknowledge several limitations of this study. Due to relatively limited sample size, further studies with larger samples are needed to validate the results. Accordingly, a wider patient demographic range could provide broader perspective. Results may vary depending on patients’ exercise routines, acute and chronic stress conditions, and lifestyle habits. The reproducibility of the results may be also restricted by the limited standardization of some of the procedures used in this study’s methodology and the lack of uniform algorithms, e.g., for tear collection. We cannot exclude the possibility that presence of a strip in the conjunctival sac might induce an adverse inflammatory response on the ocular surface. Accordingly, varying time intervals from strip insertion to its removal might have a possible impact on the obtained results. Thus, the interpretation of the data to provide predictive value for ocular surface exams must be performed with caution.

Despite these factors, our findings contribute to a better understanding of the associations between DR, DCN, and anterior-eye-surface homeostasis, thus allowing the future development of a new diagnostic and treatment path for patients with T2DM.

## 4. Materials and Methods

### 4.1. Study Group

This retrospective cohort study included 52 patients diagnosed with DR during the course of T2DM and 52 nondiabetic sex- and age-matched controls. All patients enrolled in this study completed a detailed questionnaire regarding the most common chronic diseases and related medications taken, a basic ophthalmologic health questionnaire, and the OSDI questionnaire. The general exclusion criteria included type 1 diabetes mellitus (T1DM), rheumatoid and autoimmune diseases (i.e., rheumatoid arthritis, systemic lupus erythematosus, Sjögren syndrome, or scleroderma), hyper or hypothyroidism, and active cancer during chemotherapy or radiotherapy. The ocular exclusion criteria included clinically confirmed and treated DED, a history of eye surgery or trauma within the last 3 months, contact lens use, lid and adnexal disorders, corneal pathologies, and active conjunctivitis. All participants underwent tear sampling and ophthalmological examination, which consisted of eye surface examination, fundus examination, optical coherence tomography, esthesiometry, and IVCM. Informed consent forms in accordance with the tenets of the Declaration of Helsinki were signed by all patients before study enrolment. This study was conducted according to the guidelines of the Declaration of Helsinki, and it has been approved by the Bioethics Committee of the Pomeranian Medical University (protocol code KB-006/23/2022, date of approval: 18.05.2022).

### 4.2. General Medical Assessment

All patients were interviewed and examined to collect information on weight, height, BMI, and smoking status. Study participants were also interviewed for the severity and duration of T2DM, the need for insulin therapy, and the presence of complications, such as diabetic neuropathy, diabetic kidney disease, or diabetic foot syndrome.

### 4.3. Ophthalmological Questionnaire

A basic ophthalmologic questionnaire was used to assess the patient’s current ophthalmic disorder history, e.g., glaucoma, contact lens use, past anterior eye injuries, past eye surgeries, allergic seasonal, viral/bacterial conjunctivitis, or dry eye syndrome. The OSDI score was also recorded.

### 4.4. Tear Sample Collection and Analysis

Tears were collected at the time of enrolment to assess the expression of proinflammatory cytokines and neurotrophic factors in the tear film. Schirmer’s strips (TearFloTM, HUB Pharmaceuticals, Scottsdale, AZ, USA) were used to collect the tear fluid from all subjects. Similarly, as described in our previous publications [40], the strip was placed in the lower conjunctival sac, and tears were allowed to diffuse into the strip until reaching 15 mm on the strip scale. All patients enrolled in this study were examined in the morning and were asked not to wear contact lenses, use any eye drops, or wear makeup on the day of the visit. The subjects were allowed to blink freely during this time. The Schirmer strip was then placed into an Eppendorf tube and frozen at −80 °C. Tear fluid was extracted from Schirmer’s strips by agitating small-cut pieces of these strips in 300 μL of phosphate-buffered saline. The concentrations of NGF-β, BDNF, NT-4, and inflammatory mediators (TNF-α, IL-1β, IL-2, IL-4, IL-5, IL-6, IL-8, IL-10, IL-12 p70, GM-CSF, and IFN-γ) in tear fluids were measured by multiplex fluorescent bead-based immunoassays (Luminex Corporation, Austin, TX, United States) using commercial R&D Systems Luminex Human Discovery Assay (LXSAHM) and High Sensitivity Cytokine Premixed Kit A Performance Assay (FCSTM09; R&D Systems, Minneapolis, MN, United States). A total of 50 µL of blanks, standards, and samples were added to the plate together with a microparticle cocktail and incubated in the dark for 2 h at room temperature on a horizontal orbital microplate shaker set at 800 rpm. After this step, the wells were washed with 100 µL of wash buffer three times by using a hand-held magnet. Biotin–antibody cocktail (50 µL) was added to the plate and incubated with agitation at room temperature for 1 h in the dark. After washing, 50 µL of streptavidin–PE was added to each well and incubated in the dark for 30 min on a plate shaker. Finally, after washing, the microspheres in each well were resuspended in 100 µL of wash buffer and shaken for 2 min at room temperature. The plate was then read and analyzed on an Luminex 200 analyzer, and analyte concentrations were determined from five different standard curves showing the MFI (median fluorescence intensity) vs. protein concentrations. To standardize the final concentration values, the obtained data were normalized to the total protein concentration.

### 4.5. Eye Surface Examination

The eye surface examination was conducted using an ocular surface analyzer IDRA (SBM System^®^, Torino, Italy). Analysis consisted of the assessment of interferometry; measurement of the height of the tear meniscus; NIBUT; assessment of the blinking pattern comprising the assessment of blinking frequency (the average time in seconds that elapsed between blinks); the number of full and partial blinks for 20 s; and the interblink duration measurements. Furthermore, pupillometry measurements of the pupil diameter under mesopic, scotopic, and photopic conditions; assessments of the severity of blepharitis and bulbar conjunctival redness (Cornea and Contact Lens Research Unit–CCLRU grading scale); and automated meibography were also conducted. Meibography examination consisted of semiautomated analysis of the meibomian glands located on the upper eyelid, and the percentage of meibomian gland-loss area was recorded.

### 4.6. Fundus Examination and OCT

All patients enrolled in the study group underwent fundus assessment and optical coherence tomography of the macular region to confirm or exclude macular oedema. Data regarding central retinal thickness, total macular volume, and inner (foveal) macular volume were also recorded. Data regarding patients who underwent laser PRP or intravitreal therapy with anti-VEGF injections were also collected. Additionally, to definitively grade the DR stage and rule out neovascularization in uncertain cases, fluorescein angiography was carried out. According to the fundus examination results, the patients were divided into four groups (stages 1–4) based on disease severity as determined by the International Clinical Diabetic Retinopathy Disease Severity Scale [41]. Stage 1 included patients who had mild NPDR and were diagnosed only with microaneurysms. Stage 2 included patients with moderate NPDR who had more fundus examination findings than only microaneurysms and less than those described in patients with severe disease. Stage 3 included patients with severe NPDR who had no signs of PDR and any of the following: 20 intraretinal hemorrhages in each of the 4 quadrants, venous beading in ≥2 quadrants, and/or prominent intraretinal microvascular abnormality (IRMA) in ≥1 quadrant. Stage 4 represented PDR, which was diagnosed based on the presence of neovascularization and/or vitreous or preretinal hemorrhage. All nondiabetic patients enrolled in the control group were assigned to Stage 0 according to the abovementioned severity scale, which meant that there were no abnormalities in the fundus.

### 4.7. Esthesiometry

The degree of CS in the center of the cornea and its four quadrants was assessed with a Cochet-Bonnet esthesiometer (Model L12 No. 8796, Luneau Technology, Prunay-le-Gilon, France) with a nominal 0.12 mm diameter nylon fiber. This subjective examination was performed for the functional assessment of the corneal SNP. CS thresholds were determined by recording the longest length of nylon fiber that produced a mechanical sensation of contact on the corneal surface. In addition to the assessment for individual quadrants, an average result, calculated on the basis of the results obtained in the center and 4 peripheral quadrants, was also recorded.

### 4.8. In Vivo Corneal Confocal Microscopy

IVCM (HRT3-RCM, Heidelberg Engineering, Heidelberg, Germany) was performed on the SNP in the center of the cornea. Scanning was performed in a 400 × 400 μm frame. Images from the center of the cornea were acquired by two researchers, who selected the 5 best images for further graphical and mathematical analyses. The obtained images of corneal innervation were then analyzed using ACCMetrics software (University of Manchester, Manchester, Great Britain) to assess innervation in terms of density (CNFD, [number of fibers/mm^2^]), corneal nerve fiber length (CNFL, [length of fibers/mm^2^]), corneal nerve branch density (CNBD, [number of branches/mm^2^]), corneal nerve fiber area (CNFA [mm^2^/mm^2^]), and corneal nerve fiber width (CNFW, [mm/mm^2^]). Additionally, innervation was assessed in terms of corneal nerve fiber tortuosity (CNFTo) and corneal nerve fiber reflectivity (CNFRe) based on the Oliveira-Soto scale [42].

### 4.9. Statistical Analysis

The statistical analysis was conducted using Statistica 13 software. The Mann–Whitney U test was used to compare quantitative and rank variables between groups. The strength of associations between quantitative and rank variables was measured with the Spearman rank correlation coefficient (Rs) presented with its 95% confidence interval (95% CI). Fisher’s exact test was used to compare qualitative variables between groups. Quantitative variables are presented as the mean ± standard deviation (SD) and/or median [interquartile range (IQR)]. A general linear model was used for multivariate analysis adjusted for age and sex differences between groups, with a logarithmic normalizing transformation applied to the dependent variable when needed. A *p* value of less than 0.05 was considered significant. For the purposes of statistical calculations, the average results obtained from the right and left eyes were used.

## 5. Conclusions

To the best of our knowledge, this is the first study to evaluate the associations between tear film neurotrophic and inflammatory factors, corneal innervation status, and anterior-eye-surface homeostasis in individuals with DR. The lack of a proinflammatory response in diabetic eyes and the lack of differences between groups in terms of objective clinical features indicate that the association between diabetes and DED seems to be not a simple cause-and-effect relationship. We cannot exclude the possibility that the particular techniques employed in this study might have an impact on the obtained results. Further research is required to corroborate the exact role of diabetes in development of DED. Corneal innervation disorders, which are clinically noticeable as decreased corneal sensation, result in several complications that have a direct impact on the subjective feelings of the patient. The progression of DR appears to be correlated with changes in the corneal SNP, thus emphasizing the significance of a confocal microscopic examination for DR patients.

## Figures and Tables

**Table 1:** Clinical characteristics of the study groups. Statistically significant values are shown in bold.

Parameter	Control Group	DR Group	*p* *
Age (median (IQR))	63 (12)	65 (11.5)	0.17
Sex (F/M)	27/25	17/35	0.07
BMI (median (IQR))	26.42 (5.28)	27.88 (4.69)	0.11
IOP (median (IQR))	15.5 (2.75)	15 (3.25)	0.09
BCVA (median (IQR))	1 (0.05)	0.5 (0.37)	**<0.001**
Smoking status (%)	13.46%	11.54%	1.0
HA (%)	28.85%	46.15%	0.1
Glaucoma (%)	3.85%	6%	0.67
Cataract surgery in anamnesis (%)	5.77%	10%	0.48

* Fisher exact test for qualitative variables or Mann–Whitney U test for quantitative and rank variables. IQR, interquartile range.

**Table 2:** Tear film-related factors and ocular symptoms in the DR and Control groups. Statistically significant values are shown in bold.

Parameter	Control Group Mean ± SD Median (IQR)	DR Group Mean ± SD Median (IQR)	*p* *
OSDI	11.67 ± 17.2	29.46 ± 23.95	**<0.001**
	5.5 (11.5)	27.5 (42)	
Schirmer test (mm)	15.07 ± 9.1	13.82 ± 8.2	0.42
	13 (13.75)	11 (9.25)	
NIBUT (s)	6.26 ± 2.56	6.04 ± 2.78	0.81
	5.15 (3.1)	5.75 (2.85)	
Interferometry	78.59 ± 15.45	75.69 ± 17.25	0.43
	82.25 (17.25)	77.5 (25.25)	
Tear meniscus (mm)	0.29 ± 0.12	0.36 ± 0.18	0.08
	0.26 (0.1)	0.3 (0.23)	
Bulbar redness	2.22 ± 0.67	2.44 ± 0.91	0.08
	2 (1)	3 (1)	
Blepharitis	0.5 ± 0.59	0.49 ± 0.48	0.77
	0.5 (1)	0.5 (1)	
Meibomian gland loss (%)	33.18 ± 14.09	33.7 ± 13.83	0.92
	32 (19)	31.5 (18.5)	

* Mann–Whitney U test for comparison of the 2 groups. IQR, interquartile range.

**Table 3:** Differences in subbasal nerve parameters between DR patients and controls. Abbreviations used: CNFD—corneal nerve fiber density; CNFL—corneal nerve fiber length; CNBD—corneal nerve branch density; CNFA—corneal nerve fiber area; CNFW—corneal nerve fiber width; CNFrDim—corneal nerve fractal dimension; CNFTo—corneal nerve fiber tortuosity; CNFRe—corneal nerve fiber reflectivity. Statistically significant values are shown in bold.

Parameter	Control Group Mean ± SD Median (IQR)	DR Group Mean ± SD Median (IQR)	*p* *
CNFD (no./mm^2^)	19.07 ± 6.54	9.26 ± 6.11	**<0.001**
	18.75 (8.85)	8.33 (9.11)	
CNFL (mm/mm^2^)	11.47 ± 3.17	6.7 ± 3.49	**<0.001**
	11.09 (4.56)	6.52 (5.5)	
CNBD (no./mm^2^)	22.34 ± 13.93	10.04 ± 9.79	**<0.001**
	18.75 (16.14)	7.3 (11.98)	
CNFA (mm^2^/mm^2^)	0.01 ± 0.002	0.003 ± 0.002	**<0.001**
	0.01 (0.002)	0.003 (0.003)	
CNFW (mm/mm^2^)	0.02 ± 0.001	0.02 ± 0.003	**0.02**
	0.02 (0.002)	0.02 (0.002)	
CNFrDim	1.44 ± 0.05	1.36 ± 0.09	**<0.001**
	1.45 (0.05)	1.37 (0.14)	
CNFTo	1.77 ± 1	2.21 ± 1.08	0.06
	2 (1.5)	2 (1.5)	
CNFRe	3.3 ± 0.85	2.43 ± 1.15	**<0.001**
	3.5 (1.25)	2.5 (1.5)	

* Mann–Whitney U test for comparison of the 2 groups. IQR, interquartile range.

**Table 4:** Tear film cytokine levels among DR patients and control subjects (pg/mL). Statistically significant values are shown in bold.

Parameter	Control Group Mean ± SD Median (IQR)	DR Group Mean ± SD Median (IQR)	p*
TNF-α	4.77 ± 1.84	3.87 ± 2.14	**0.003**
	4.61 (2.45)	3.19 (2.36)	
VEGF	185.32 ± 79.45	160.31 ± 62.22	0.13
	158.24 (112.32)	152.39 (69.63)	
IL-2	1.23 ± 0.7	0.88 ± 0.46	**0.002**
	1.14 (0.79)	0.83 (0.38)	
IL-1β	14.58 ± 24.09	10.87 ± 11.79	0.48
	8.49 (10.06)	6.62 (8.36)	
IL-4	44.59 ± 18.55	43.34 ± 19.33	0.53
	39.97 (21.38)	39.79 (16.02)	
IL-5	0.53 ± 0.35	0.48 ± 0.23	0.86
	0.44 (0.24)	0.44 (0.26)	
IL-6	3.05 ± 3.17	4.03 ± 3.66	0.3
	2.32 (2.23)	3.11 (4.33)	
IL-8	504.83 ± 389.24	377.97 ± 316.71	**0.03**
	383.89 (339.09)	277.4 (343.47)	
IL-10	1.53 ± 0.74	1.14 ± 0.64	**0.0003**
	1.36 (0.79)	1.03 (0.44)	
IL-12	11.67 ± 8.1	16.24 ± 23.64	0.07
	9.73 (4.68)	11.64 (7.12)	
GM-CSF	2.6 ± 1.58	2.06 ± 1.33	**0.03**
	2.14 (1.68)	1.74 (1.22)	
IFN-γ	1.89 ± 2.5	0.7 ± 0.97	**0.002**
	1.21 (2.26)	0.2 (1.13)	

* Mann–Whitney U test for comparison of the 2 groups. IQR, interquartile range.

**Table 5:** Tear film neurotrophic factor levels among DR patients and control subjects (pg/mg). Statistically significant values are shown in bold.

Parameter	Control Group Mean ± SD Median (IQR)	DR Group Mean ± SD Median (IQR)	*p* *
BDNF	2.29 ± 0.8	1.82 ± 0.89	**0.0001**
	2.17 (0.97)	1.43 (0.82)	
NGF-β	1.04 ± 0.68	1.35 ± 0.97	0.10
	0.90 (0.58)	0.97 (0.85)	
NT-4	6.55 ± 2.75	7.84 ± 3.67	0.06
	6.15 (3.14)	6.98 (3.03)	

* Mann–Whitney U test for comparison of the 2 groups. IQR, interquartile range.

## Data Availability

The data that were used to support the findings of this study are available from the corresponding author upon request.
